# Assessing the acoustic behaviour of *Anopheles gambiae* (*s.l.*) *dsxF* mutants: implications for vector control

**DOI:** 10.1186/s13071-020-04382-x

**Published:** 2020-10-07

**Authors:** Matthew P. Su, Marcos Georgiades, Judit Bagi, Kyros Kyrou, Andrea Crisanti, Joerg T. Albert

**Affiliations:** 1 Ear Institute, University College London, 332 Grays Inn Road, London, WC1X 8EE UK; 2 The Francis Crick Institute, 1 Midland Road, London, NW1 1AT UK; 3 Division of Biological Science, Nagoya University, Nagoya, 464-8602 Japan; 4 Department of Life Sciences, Imperial College London, London, UK

**Keywords:** *Anopheles gambiae* (*s.l.*), *Anopheles coluzzii*, *Doublesex*, Gene drive, Wing beat frequency, Flight tone, Mosquito, Acoustic communication, Hearing, Phonotaxis, Vector control

## Abstract

**Background:**

Release of gene-drive mutants to suppress *Anopheles* mosquito reproduction is a promising method of malaria control. However, many scientific, regulatory and ethical questions remain before transgenic mosquitoes can be utilised in the field. At a behavioural level, gene-drive carrying mutants should be at least as sexually attractive as the wildtype populations they compete against, with a key element of *Anopheles* copulation being acoustic courtship. We analysed sound emissions and acoustic preference in a *doublesex* mutant previously used to collapse *Anopheles gambiae* (*s.l.*) cages.

**Methods:**

*Anopheles* rely on flight tones produced by the beating of their wings for acoustic mating communication. We assessed the impact of disrupting a female-specific isoform of the *doublesex* gene (*dsxF*) on the wing beat frequency (WBF; measured as *flight tone*) of males (XY) and females (XX) in homozygous *dsxF*^*−*^ mutants (*dsxF*^*−/−*^), heterozygous *dsxF*^*−*^ carriers (*dsxF*^+/−^) and G3 *dsxF*^+^ controls (*dsxF*^+*/*+^). To exclude non-genetic influences, we controlled for temperature and wing length. We used a phonotaxis assay to test the acoustic preferences of mutant and control mosquitoes.

**Results:**

A previous study showed an altered phenotype only for *dsxF*^*−/−*^ females, who appear intersex, suggesting that the female-specific *dsxF* allele is haplosufficient. We identified significant, dose-dependent increases in the WBF of both *dsxF*^*−/−*^ and *dsxF*^+/−^ females compared to *dsxF*^+*/*+^ females. All female WBFs remained significantly lower than male equivalents, though. Males showed stronger phonotactic responses to the WBFs of control *dsxF*^+*/*+^ females than to those of *dsxF*^+/−^ and *dsxF*^*−/−*^ females. We found no evidence of phonotaxis in any female genotype. No male genotypes displayed any deviations from controls.

**Conclusions:**

A prerequisite for anopheline copulation is the phonotactic attraction of males towards female flight tones within mating swarms. Reductions in mutant acoustic attractiveness diminish their mating efficiency and thus the efficacy of population control efforts. Caged population assessments may not successfully reproduce natural mating scenarios. We propose to amend existing testing protocols to better reflect competition between mutants and target populations. Our findings confirm that *dsxF* disruption has no effect on males; for some phenotypic traits, such as female WBFs, the effects of *dsxF* appear dose-dependent rather than haplosufficient.
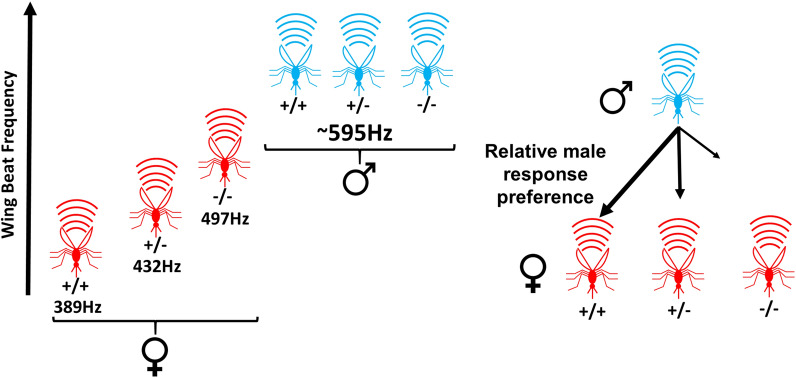

## Background

Mosquitoes represent a major global health problem, with *Aedes*, *Anopheles* and *Culex* species acting as vectors of diseases that infect millions of people each year [1]. Malaria remains a major cause of mortality and morbidity worldwide despite significant advances made in disease control since the turn of the century [2, 3]. This is in part due to the reduced efficacy of current control tools such as insecticidal nets and indoor residual spraying, as well as the emergence of secondary disease vectors [4–6]. Novel control techniques are therefore necessary to continue the push towards disease elimination [7].

One potential option is the utilisation of gene drive systems, which target haplosufficient female fertility genes, leading to a reduction in female fertility and, eventually, population collapse [8, 9]. The recent generation of *Anopheles gambiae* CRISPR/Cas9 mutants in which a female specific exon of the *doublesex* (*dsxF*) gene was disrupted is here of interest. Laboratory cage trials have demonstrated that the introduction of *dsxF* mutants into cages of wildtype mosquitoes was sufficient to lead to eventual population collapse [10].

However, there are many scientific, ethical and regulatory hurdles to overcome before such transgenic mosquitoes can be released in even semi-field trials [11]. It is vital that any transgenic mosquitoes are subjected to rigorous testing prior to use in the field; gene transfer into natural populations following release of transgenic *Aedes aegypti* has highlighted the potential risks of release of transgenic insects [12]. On a scientific level, one important task will be to maintain the gene drive’s effectiveness outside of the laboratory and under more ‘real world’ scenarios.

A major element of this testing is the investigation of interactions with natural, non-mutant populations, particularly with regards to courtship behaviour. If mutant mosquitoes are unable, or only less likely, to copulate with native populations then they become the less attractive option, which will slow down or outright frustrate the population control effort [13]. In addition to potential direct and indirect fitness costs associated with mutations, laboratory habituation and mass rearing can also affect mating performance [14, 15]. In this context it is noteworthy that the *dsxF* mutants were generated from a laboratory-established strain (G3) rather than any wildtype population [10]. Extensive testing of mutant mating fitness prior to translation from laboratory mating assays is thus a key requirement for assessing a specific line’s suitability for use as part of a release programme.

The sense of hearing is a vital component of mosquito reproduction, with males identifying females within swarms *via* phonotactic responses to female flight tones and acoustic communication is also thought to play a role in female mate selection [16–19]. The phonotactic response is highly specific, however, with males responding only to a narrow range of frequencies [20]. Both male and female mosquitoes have extraordinarily sensitive and complex ears, but there are also significant sexual dimorphisms in auditory function and hearing-related behaviours [21–23].

Chromosomally female (XX) *dsxF*^*−/−*^ mutants display an intersex phenotype, which also includes an intersex morphology of their flagellar sound receivers [10]; if, and if so to what extent, other parts of the auditory or acoustic system are affected by the allelic disruption is unclear. Physiological changes that could impact the mutants’ ability to interbreed with existing mosquitoes are, e.g. changes in male or female flight tones or their corresponding acoustic preferences. It is currently unclear if any of the *dsxF* mutant genotypes affects these parameters. If so, this could have substantial effects on the ability of mutants to interbreed with existing mosquitoes.

In order to address this topic, we tested the flight tones and phonotactic responses of *dsxF* XX and XY mutants and controls. We found that whilst male (XY) mutant (*dsxF*^*−/−*^, *dsxF*^±^) flight tones were not significantly different to male controls (*dsxF*^+*/*+^), female (XX) mutant (*dsxF*^*−/−*^, *dsxF*^+/−^) flight tones had significantly higher frequencies than those of their respective controls (*dsxF*^+*/*+^), with both showing an increase towards the male flight tone in a seemingly dose-response fashion.

No female showed evidence of phonotaxis to any of the acoustic stimuli we provided, whilst all males showed a strong phonotactic response to tones of 400 Hz (but much reduced or absent responses to tones of 100 Hz or 700 Hz). However, a more focused phonotaxis assay using the median flight tones obtained from each of the three female genotypes (*dsxF*^+*/*+^, *dsxF*^+/−^, *dsxF*^*−/−*^) found that control males responded far more strongly to the flight tones of control females than to either of the mutant flight tones. Preliminary tests of *dsxF*^*−/−*^ males showed a similar preference for control flight tones (Additional file 1: Figure S1). As such, it seems likely that male mosquitoes of any genotype will demonstrate a strong preference for wildtype females, with mutant females potentially reduced to a lesser attractive role.

## Methods

### Mosquito rearing

*Anopheles gambiae* G3 strain (*dsxF*^+/+^), as well as *dsxF*^+/−^ and *dsxF*^−/−^ mutant pupae, were reared and provided by the Crisanti Lab at Imperial College London, UK. Larval density was kept constant throughout the rearing process.

*dsxF*^+/+^ and *dsxF*^+/−^ pupae were sex separated and kept in single sex cages in incubators maintained at 28 °C and 80% relative humidity. Light/dark conditions included a one-hour ramping period of constantly increasing white light; ramp for lights-ON from zeitgeber time (ZT) ZT0 to ZT1; then 11 h (ZT1-ZT12) of white light at constant intensity, followed by a one-hour (ZT12-13) ramping period of constantly decreasing white light, and then 11 h (ZT13-ZT24) of constant darkness. All light ramps transitioned linearly between a Photosynthetic Photon Flux Density (PPFD) of 80 µmol/m^2^/s (or ~ 5929 lx) and complete darkness (0 µmol/m^2^/s or 0 lx, respectively).

*dsxF*^*−/−*^ pupae were not separated by sex but were otherwise reared in identical conditions. Mosquitoes were supplied with a constant source of 10% glucose solution.

All mosquitoes used for experiments were virgin and aged 3–7 days-old. Mosquitoes were housed within temperature, humidity and light-controlled incubators for three days prior to all experiments, which were conducted during a time corresponding to sunset (which represents a time period of peak activity and swarming under natural conditions).

### Wing length measurements

The right wings of adult mosquitoes from each genotype were removed using a pair of forceps whilst the mosquitoes were CO_2_ sedated. The wings and flagellae were then transferred to separate microscope slides in groups of five. Each individual sample was immediately imaged using a Zeiss Axioplan 2 microscope and Axiovision 4.3 software. Wing lengths were determined using the Axiovision 4.3 software length measurement function, calibrated to the nearest 0.1 mm. Three biological repeats were conducted over separate generations.

Total sample sizes for each group: *dsxF*^+*/*+^ XX = 40; *dsxF*^+/−^ XX = 40; *dsxF*^*−/−*^ XX = 40; *dsxF*^+*/*+^ XY = 41; *dsxF*^+/−^ XY = 40; *dsxF*^*−/−*^ XY = 41.

### Wing beat frequency measurements

A resin casing was printed using an Ultimaker 2 + 3D printer and used to house a particle velocity microphone (Knowles NR-3158). The whole apparatus was held in a micromanipulator placed on a vibration isolation table.

Adult mosquitoes from each genotype were cold-sedated using ice before blue-light cured glue was used to fix the tip of a tungsten wire to their thoraces, taking care not to restrict or damage the wings in doing so. The tethered mosquito was mounted into the microphone case and oriented such that its posterior was facing the particle velocity microphone. All measurements were conducted in the same isolated room at a temperature between 21–22 °C.

Mosquito flight was initiated *via* a tarsal reflex response [24]. A small cotton ball was placed underneath each tethered mosquito; once the mosquito had clasped the ball, it was swiftly removed, with this removal stimulating flight initiation. Minimum flight length used was 10 s. The voltage timeseries waveform measured for each flying mosquito by the particle velocity microphone was recorded using the Spike2 software (Cambridge Electronic Design Ltd., UK).

Sample sizes for each group were: *dsxF*^+*/*+^ XX = 30; *dsxF*^+/−^ XX = 30; *dsxF*^*−/−*^ XX = 30; *dsxF*^+*/*+^ XY = 27; *dsxF*^+/−^ XY = 30; *dsxF*^*−/−*^ XY = 30.

### Flight tone analysis algorithm

Raw data from the Spike2 recordings were exported to Python for analysis *via* a custom script. The first and last two seconds of each flight were discarded prior to analysis. Subsequently the timeseries was divided into 5-s subsegments, discarding the final shorter subsegment (if flight length modulo 5 ≠ 0). A Fast Fourier transform (FFT) with a 200 ms window was then applied throughout each of the subsegments. This window was shifted in 100 ms increments (i.e. 50% overlap between successive FFTs) and applied repeatedly until the end of the flight segment was reached.

Limits were applied to the frequency domain of each FFT such that only frequencies between 200–1000 Hz would be extracted for analysis. For each FFT, the peak frequency was identified and assigned as the flight tone for the time segment over which the FFT was calculated. A list of peak frequencies was compiled for each of the aforementioned subsegments. These lists were added together and averaged, resulting in a 5-s long final list of average frequencies. The mean was employed in the averaging step as these values were normally distributed. As the list of means, in turn, tended to be non-normally distributed, the median was taken and assigned as the flight tone of that individual animal. The segmentation of the original waveform and summarization into a single 5-s long list of values served to moderate for potential effects of flight duration on the animals’ flight tone.

### Spectrally broad phonotaxis assay

Female (XX) and male (XY) mosquitoes from all three genotypes were aspirated into small, single-sex cages in groups of 25 and kept for at least two hours in the same room used for flight tone experiments. All experiments were conducted at a temperature between 20–23 °C and at ~ ZT13 (i.e. swarming time, around the time of complete cessation of light). Throughout the experiment, mosquitoes were kept in constant darkness.

A free app (TMsoft tone generator) was used to provide acoustic stimulation to caged mosquitoes; this stimulation consisted of three pure tones with frequencies of 100, 400 and 700 Hz. These frequencies were chosen based on the prior recordings which found no female flight tones as high as 700 Hz, and no male or female frequency as low as 100 Hz.

The sound source was placed next to the cage with its speaker touching the cage mesh prior to stimulus initiation. Each tone was played for 1 min and was succeeded in turn by a 1-min long silence before the next tone was played. The tones were played first from low to high frequencies and subsequently from high to low, allowing mosquitoes to rest for 5 min between forward and backward playbacks. To ensure that mosquitoes were being attracted to the sound emitted by the sound source rather than the sound source itself, at the start of each experiment the sound source was placed next to the cage with its speaker touching the cage’s mesh with no stimulus playing. Mosquitoes that approached the sound source during either control or acoustic playback were counted manually using a red-light flashlight. Three biological repeats were conducted for each group.

### Spectrally focused phonotaxis assay

*dsxF*^+*/*+^ XY mosquitoes were tested in groups of 25 as above for the broad-range assay, this time, however using three pure tones with frequencies equal to the recorded median flight tone frequencies of each of the female genotypes; 380 Hz (*dsxF*^+*/*+^), 432 Hz (*dsxF*^+/−^) and 497 Hz (*dsxF*^*−/−*^). [Please note that the played *dsxF*^+*/*+^ control tone of 380 Hz is marginally different from the median WBF calculated for *dsxF*^+*/*+^; this is because the flight tone choice for the playback experiments was based on an earlier data cohort].

### Statistical analysis

Flight tone analyses were conducted in Python. Remaining analyses were completed in Matlab and R. Throughout the analyses, all statistical tests used a significance level of *P* < 0.05.

Sample sizes for all experiments were determined *via* reference to published investigations. Within-group variation estimates were calculated when appropriate as part of standard statistical testing.

Statistical tests for normality (Shapiro–Wilk Normality tests with a significance level of *P* < 0.05) were first applied to each dataset. Wing length measurements and flight tones were found to be normally distributed; two-way ANOVA tests were thus used for comparisons across the genotypes and sexes.

For the spectrally broad phonotaxis assay, the proportion of responders to the control stimulus (silence) was subtracted from the proportion of responders to the stimulus tones. That is, to calculate the adjusted proportion of responders we calculated:

$${\text{Adjusted}}\;{\text{proportion}}\;{\text{of}}\;{\text{responders}} = \frac{{{\text{Responder}}{{\text{s}}_{tone}} - {\text{Responder}}{{\text{s}}_{silence}}}}{{{\text{Total}}\;{\text{number}}\;{\text{of}}\;{\text{mosquitoes}}}}$$

where Responders_*tone*_ and Responders_*silence*_ refers to the number of responders to the individual tones or silence respectively. One-way ANOVAs were then used to test for differences in responses between the stimulus tone frequencies. For the focused phonotaxis assay, no adjusted proportion was calculated, and one-way ANOVAs were applied directly to the proportion of responders.

## Results

### *dsxF*^+/−^ and *dsxF*^*−/−*^ XX mutants have different flight tones to all other XX and XY mosquitoes

**Fig. 1:**
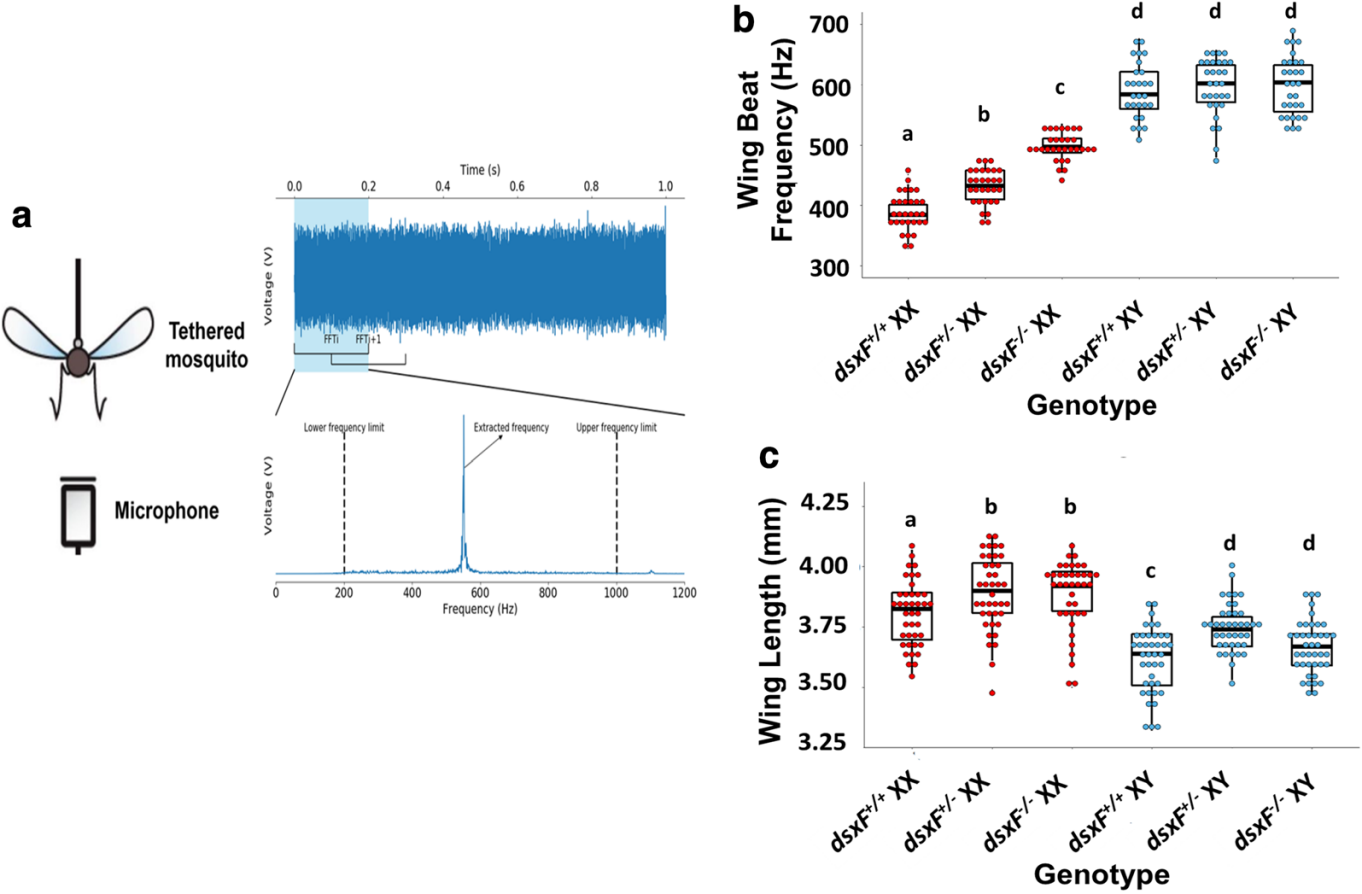
dsxF^+/−^ and dsxF^−/−^ XX mutants have different wing beat frequencies (= flight tones) to all other groups. **a** Sketch of flight tone recording set-up: mosquitoes were tethered then placed at a constant distance from a microphone. Temperature and humidity conditions were controlled (21–22 °C; 50% RH) and recordings always took place within the same two-hour window. **b** Calculated wing beat frequencies for each genotype. Significant differences (two-way ANOVA; *P < 0.05) between groups are indicated by letter. Centre line mean; box limits, lower and upper quartiles; whiskers, 5th and 95th percentiles (identical B-C). Sample sizes: *dsxF*^+*/*+^ XX = 30; *dsxF*^+/−^ XX = 30; *dsxF*^*−/−*^ XX = 30; *dsxF*^+*/*+^ XY = 27; *dsxF*^+/−^ XY = 30; *dsxF*^*−/−*^ XY = 30. **c** Wing length measurements for each genotype. Significant differences (two-way ANOVA; *P < 0.05) between groups are indicated by letter. Sample sizes: *dsxF*^+*/*+^ XX = 40; *dsxF*^+/−^ XX = 40; *dsxF*^*−/−*^ XX = 40; *dsxF*^+*/*+^ XY = 41; *dsxF*^+/−^ XY = 40; *dsxF*^*−/−*^ XY = 41

By recording the flight tones of tethered female and male mosquitoes (Fig. 1a), we were able to calculate the median flight tones for each group (Fig. 1b). All male flight tones were found to be greater than all female flight tones (ANOVA: *F*_(1, 114)_ = 336.471, *P* < 0.0001), but we found no differences between males (ANOVA: *F*_(2, 57)_ = 0.51354, *P* = 0.6011). The flight tones of *dsxF*^*−/−*^ females were significantly different from all other groups; they were significantly higher than the other female genotypes (497 ± 22.2 Hz compared to 432 ± 28.7 Hz and 380 ± 30.0 Hz for *dsxF*^+/−^ and *dsxF*^+*/*+^, respectively; ANOVA: *F*_(2, 57)_ = 75.384, *P* < 0.0001), and significantly lower than all male genotypes (ANOVA: *F*_(1, 114)_ = 336.471, *P* < 0.0001; Table 1; Fig. 1b). We also found a significant difference between *dsxF*^+/−^ XX mutants and the other two female genotypes in an apparent dose response fashion (ANOVA: *F*_(2, 57)_ = 75.384, *P* < 0.0001; Fig. 1b).

**Table 1:** Quantification of changes to *dsxF*^+/−^ XX flight tones

	*dsxF*^+*/*+^ XX	*dsxF*^+/−^ XX	*dsxF*^*−/−*^ XX	*dsxF*^+*/*+^ XY	*dsxF*^+/−^ XY	*dsxF*^*−/−*^ XY
Sample size, wing length	40	40	40	41	40	41
Wing length in mm (SD)	3.806* (0.126)	3.894 (0.152)	3.893 (0.157)	3.604*** (0.153)	3.746*** (0.100)	3.666*** (0.109)
Sample size, flight tone	30	30	30	27	30	30
Flight tone in Hz (SD)	388.52*** (29.97)	431.55*** (28.68)	497.18 (22.22)	590.60*** (46.10)	596.14*** (47.40)	597.71*** (47.33)

*Notes*: Mean values of wing lengths and flight tones for *dsxF*^+*/*+^, *dsxF*^+/−^ and *dsxF*^+*/*+^ XX and XY mosquitoes, with standard deviation (SD) values provided in brackets. Significant differences found between *dsxF*^*−/−*^ XX mosquitoes and any other mosquito group in terms of wing length (ANOVA: males, *F*_(1, 114)_ = 82.7644, *P* < 0.0001; *dsxF*^+*/*+^ XX, *F*_(2, 57)_ = 3.731, *P* = 0.01) or flight tone (ANOVA: *F*_(5, 114)_ = 336.471, *P* < 0.0001) are starred

Mosquito flight tone frequencies have been reported to show correlations with temperature (see, e.g. [25]. for *Aedes*), but the relation, especially for anopheline mosquitoes, has remained unclear. Here, temperature was tightly controlled, with all recordings being made between 21 and 22 °C. The relationship between wing beat frequency (= flight tone) and wing length is far more contentious however, with conflicting reports on potential correlations (see, e.g. [26, 27]). We measured wing lengths for each group and found significant differences between the sexes (ANOVA: *F*_(1, 114)_ = 82.7644, *P* < 0.0001; Fig. 1c; Table 1). Further differences were found between *dsxF*^+/−^ and *dsxF*^+*/*+^, as well as *dsxF*^*−/−*^ and *dsxF*^+*/*+^, mosquitoes of both sexes (ANOVA: males, *F*_(2, 57)_ = 5.9436, *P* = 0.0045; females, *F*_(2, 57)_ = 10.731, *P* = 0.0001). Individual correlation analyses for each group showed a relationship between wing length and wing beat frequency only for *dsxF*^+*/*+^ females (Additional file 2: Figure S2). Furthermore, a linear model fit including data from all groups found no significant relationship between wing length and wing beat frequency (see Additional file 3: Table S1).

### Male, but not female, mosquitoes show positive phonotactic responses to acoustic stimuli mimicking female flight tones

**Fig. 2:**
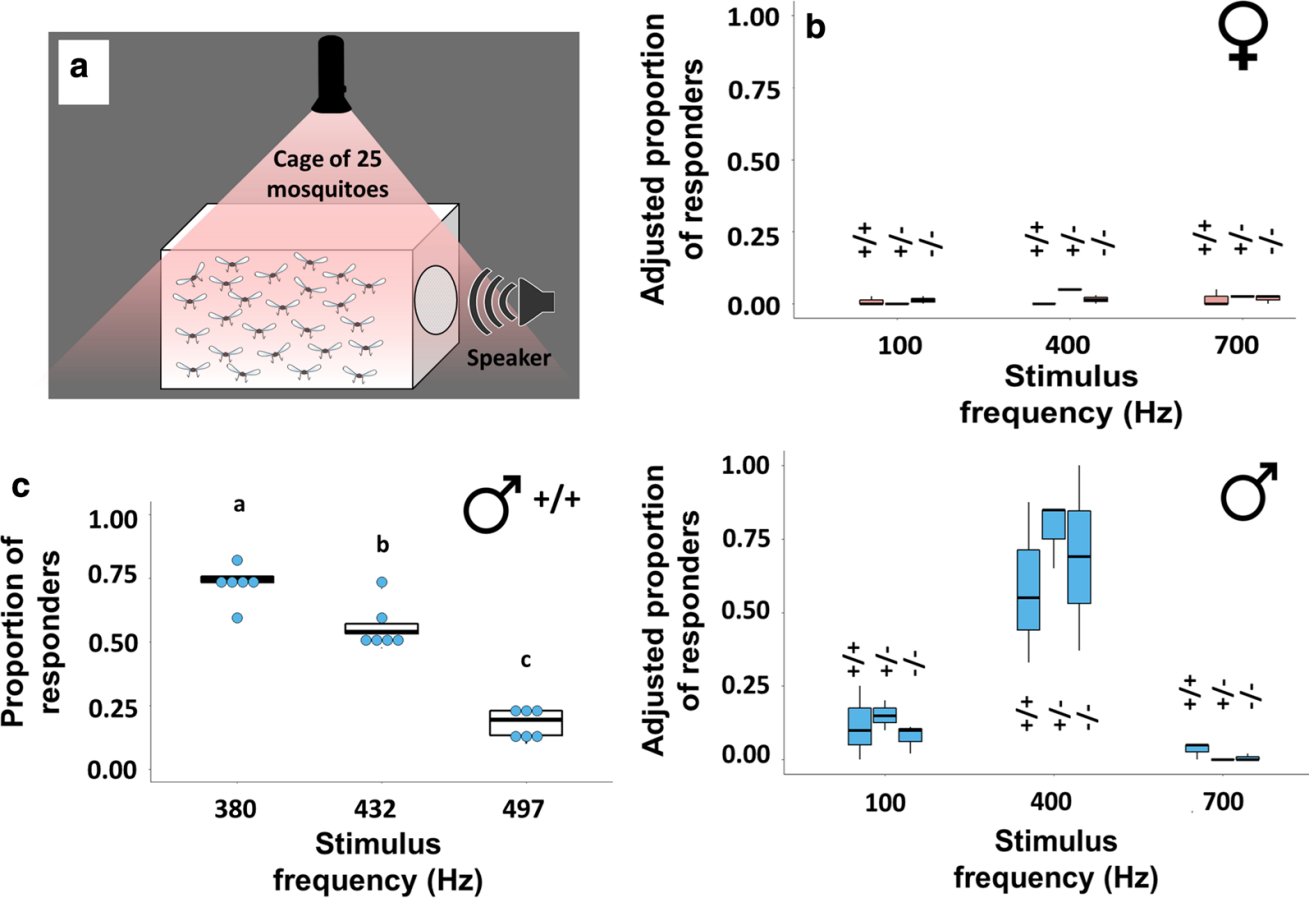
Males show a strong preference for acoustic stimuli of similar frequency to wildtype female flight tones; this phonotactic response is reduced as the tone becomes increasingly different. **a** Diagram of phonotaxis experimental set-up: Single-sex virgin cages were provided with one-minute periods of stimulation in the form of three pure tones (100, 400 and 700 Hz) or a one-minute period of silence. The number of mosquitoes attracted to the sound source for each type of stimulus was calculated. **b** Adjusted proportion of mosquitoes responding to each stimulus type (no stimulus, 100 Hz, 400 Hz and 700 Hz, respectively) for XX and XY mosquitoes from each genotype. Centre circle, median; error bars represent ± SEM. **c** Adjusted proportion of control mosquitoes responding to each stimulus type (380 Hz, 432 Hz and 497 Hz, respectively) for *dsxF*^+*/*+^ XY mosquitoes. Centre line, median; error bars represent ± SE

We tested for mosquito responses to auditory stimulation in order to investigate whether mutants showed altered behaviour (Fig. 2a). No females from any genotype showed a significantly greater response to an acoustic stimulus (defined as an approach to the sound source) than to silence (ANOVA: *F*_(2, 21)_ = 0.53743, *P* = 0.5921; Fig. 2b top; Table 2). All male groups tested were found to respond more strongly to tones of 400 Hz, the stimulus which most closely mimicked wildtype female WBF, than any other stimulus type (ANOVA: *F*_(2, 24)_ = 55.537, *P* < 0.0001; Fig. 2b bottom; Table 2). However, a few males also responded to the 100 Hz and 700 Hz tones. It seems noteworthy that the males’ flight-mediated responses to the playback tones were equally strong in mutants and controls, suggesting that the *dsxF*^*−/−*^ allele does not affect male flight behaviour (ANOVA: *F*_(4, 18)_ = 0.99707, *P* = 0.4346; Table 2).

**Table 2:** Quantification of phonotactic responses to acoustic stimulation (coarse)

Genotype	*dsxF*^+*/*+^ XX	*dsxF*^+/−^ XX	*dsxF*^*−/−*^ XX	*dsxF*^+*/*+^ XY	*dsxF*^+/−^ XY	*dsxF*^*−/−*^ XY
Sample size	3 cages of 25	2 cages of 25	3 cages of 25	3 cages of 25	3 cages of 25	3 cages of 25
Proportion of responders to control	0.02 (0.02)	0.1 (0)	0 (0)	0 (0)	0 (0)	0 (0.01)
Proportion of responders to 100 Hz	0 (0.03)	0.09 (0.01)	0 (0.01)	0.1 (0.03)*	0.15 (0.03)*	0.1 (0.08)*
Proportion of responders to 400 Hz	0.05 (0.02)	0.11 (0.04)	0 (0)	0.69 (0.18)	0.85 (0.07)	0.55 (0.17)
Proportion of responders to 700 Hz	0.05 (0.03)	0.09 (0.04)	0 (0.02)	0 (0.01)*	0 (0)*	0.05 (0.02)*

*Note*: Median values of the number of responders to coarse phonotactic stimulation for *dsxF*^+*/*+^, *dsxF*^+/−^ and *dsxF*^*−/−*^ XX and XY mosquitoes, with SEM values provided in brackets. Significant differences found within a genotype between the response to 400 Hz and 100/700 Hz stimulation are starred (ANOVA: *F*_(2,24)_ = 55.537, *P* < 0.0001)

We then investigated if the flight tone differences observed between females with different allelic combinations of *dsxF* (^+*/*+^, ^+/−^, ^*−/−*^) were behaviourally relevant. Specifically, we tested the phonotactic preferences of *dsxF*^+*/*+^ males to pure tones with frequencies equivalent to the median frequencies of females from all three genotypes (^+*/*+^  = 380 ± 30.0 Hz, ^+/−^  = 432 ± 28.7 Hz and ^+/−^  = 497 ± 22.2 Hz), at the narrow temperature range of 21–22 °C. Males were found to respond significantly more to tones similar to ‘wildtype’ *dsxF*^+*/*+^ female flight tones than to tones mimicking either of the female mutants. The ability of flight tones to induce male phonotaxis followed a ‘dose-dependent’ pattern with *dsxF*^+*/*+^  > *dsxF*^+/−^  > *dsxF*^*−/−*^ (ANOVA: *F*_(2, 15)_ = 51.122, *P* < 0.0001; Fig. 2c; Table 3).

**Table 3:** Quantification of phonotactic responses to acoustic stimulation (focused)

Genotype	*dsxF*^+*/*+^ XY
Sample size	6 cages of 25
Proportion of responders to 380 Hz	0.75 (0.03)
Proportion of responders to 432 Hz	0.54 (0.03)***
Proportion of responders to 497 Hz	0.20 (0.02)***

*Note*: Median values of the number of responders to focused phonotactic stimulation for *dsxF*^+*/*+^ XY mosquitoes, with SEM values provided in brackets. Significant differences found between the three stimulation frequencies are starred (ANOVA: *F*_(2, 15)_ = 51.122, *P* < 0.0001)

## Discussion

Hearing plays a crucial role in mosquito copulation [28]. The phonotactic responses of mosquito males to the flight tones of nearby flying females (or to artificial pure tones mimicking such females), are an important behavioural feature for mosquito reproductive fitness, and reproduction [29]. As such, the ‘acoustic fitness’ of transgenic lines marked for release in the field is a key requirement for the successful spread of deleterious mutations into wildtype populations. Here we show that the transgenic disruption of a female-specific isoform of the sex-determination gene *doublesex* (*dsxF*) changes female flight tones and that mutant flight tones elicit substantially reduced phonotactic responses in control males. The flight tone changes observed were more pronounced in homozygous (*dsxF*^*−/−*^) than in heterozygous condition (*dsxF*^*−/−*^), indicating a *dsxF*^+^ dose-dependence of this phenotypic trait, which contrasts with the previously shown haplosufficiencies [10].

Previous recordings of mosquito flight tones have implemented a variety of analytic techniques, but rarely implemented strict environmental controls. This is problematic given the significant variation for reported flight tones at different temperatures, and also the suggested correlations of flight tone and body size [25, 28]. Here we strictly controlled temperature, and also measured wing length as a proxy for body size, to control for this variability. *Anopheles* swarms form predominantly at dusk, when both light and temperature decrease rapidly [30]. It seems possible that during this time female *Anopheles* flight tones decrease rapidly in direct correlation to these temperature decreases; female *Ae. aegypti* WBF fell by around 10 Hz per degree over similar temperature changes [25]. Given the sizeable differences we observed in male phonotactic responses to acoustic stimuli less than 50 Hz apart, these differences could have a significant effect on male auditory behaviours.

If *dsxF* mutants are to be released in the wild, then only heterozygous males are likely to be released. Updated cage trials with a starting allelic frequency of only 2.5% predicted population collapse within 14 generations [31]. Generation of *dsx* mutants in other mosquito species (such as *Ae. aegypti*) could not only provide a promising control method to combat other mosquito populations, but also provide an ideal tool to investigate the fundamental mechanisms which underly the sizeable sexual dimorphisms in mosquito auditory systems and behaviours. The *dsxF* isoform is reported to be female-specific, it is therefore reassuring that we found no differences in flight tones between male genotypes. All males not only displayed typical phonotactic behaviour but furthermore retained their acoustic preference for the flight tones of control (‘wildtype’) females around 400 Hz (at 20–21 °C). Most interestingly also, the intersex phenotype of *dsxF*^*−/−*^ females did not include the display of phonotactic behaviour, possibly indicating an independence of male phonotaxis from the *dsx* pathway or leaving a role for the male *doublesex* isoform (*dsxM*).

Laboratory-based assays in cage conditions can only partially, at best, replicate field conditions. Throughout our phonotaxis experiments, we provided only a single, monofrequent acoustic stimulus at any one time. This is a poor simulation of the auditory landscape of an *An. gambiae* swarm containing many hundreds of males whose flight tones may be constantly modulated [25]. The presence of multiple females within this environment may lead to selection choices for individual males. This may exacerbate the phonotactic preferences we discovered (see Fig. 2c), with males possibly disregarding the flight tones of mutant females if simultaneously presented with the sounds of wildtype ones.

Yet the fact that mutant males retain a strong preference for the sounds of wildtype females, bodes well for the effective spread of mutant alleles into resident wildtype populations. It remains to be seen though whether mutant males can successfully join the natural swarms, in which *Anopheles* copulation occurs. Further studies of both mutant and wildtype swarming behaviour are necessary to better understand—and predict—the relevant male–female interactions. This also holds true for a potential female choice element; although we here found no differences between the male genotypes in terms of flight tones, there may be other differences which influence female mate selection.

The argument for utilising transgenic *Anopheles* strains for fighting malaria grows stronger with each new report of insecticide resistance or change in biting behaviour. It is essential however that transgenic lines are tested thoroughly for their suitability. Not only will such experimental testing improve a respective line’s chances of success, but it will also help to create a more detailed profile of the specific requirements for successful release lines (e.g. gene drive carriers). Given the importance of audition for all disease-transmitting mosquito species, acoustic (and auditory) fitness will feature high on that list of requirements. Acoustic courtship in *Anopheles*, finally, is inextricably linked to the mating swarm. Including swarming behaviour in the pre-release testing will thus be crucial. A pipeline of testing focused on mosquito acoustic mating behaviour could significantly help in boosting the efficacy of any release effort. This testing could comprise a sequence of analyses, covering anatomical investigation of the ear, functional tests of hearing, flight tone recordings, and phonotaxis/mating assays under cage or semi-field conditions. This study utilised only a fraction of these analyses and discovered ecologically relevant differences between mutant and control lines; a comprehensive assessment may provide substantially more evidence which can inform the decision-making process over mutant release strategies and help optimise future disease-control efforts.

## Supplementary information

**Additional file 1: Figure S1.** Phonotactic response of *dsxF*^−/−^ males to phonotactic stimulation.Adjusted proportion of control mosquitoes responding to each stimulus type (380 Hz, 432 Hz and 497 Hz, respectively) for *dsxF*^−/−^ XY mosquitoes. Centre line, median; error bars represent ± SE.

**Additional file 2: Figure S2.** Correlations between wing length and wing beat frequency. Correlations between wing length (mm) and wing beat frequency (Hz) for all groups tested. Sample sizes are the same as for wing beat frequency calculations.

**Additional file 3: Table S1.** Outputs of linear model relating wing beat frequency to sex, genotype and wing length. Parameter estimates for linear model fitted in R using the *lme4* package as: Wing beat frequency ~ Sex*Genotype + Wing length. Significant values are italicised. Whilst we found that sex, genotype, and sex:genotype were all highly significant factors in determining wing beat frequency, wing length was not found to significantly affect wing beat frequency.

## Data Availability

All data analysed in this paper are available from the authors, as well as more comprehensive details on experimental or analytical methodologies.

## References

[1] James SL, Abate D, Abate KH, Abay SM, Abbafati C, Abbasi N (2018). Global, regional, and national incidence, prevalence, and years lived with disability for 354 diseases and injuries for 195 countries and territories, 1990–2017: a systematic analysis for the Global Burden of Disease Study 2017. Lancet.

[2] O’Meara WP, Mangeni JN, Steketee R, Greenwood B (2010). Changes in the burden of malaria in sub-Saharan Africa. Lancet Infect Dis.

[3] WHO. Malaria Fact Sheet (https://www.who.int/en/news-room/fact-sheets/detail/malaria). https://www.who.int/en/news-room/fact-sheets/detail/malaria. Geneva: World Health Organization; 2019.

[4] Ranson H, N’Guessan R, Lines J, Moiroux N, Nkuni Z, Corbel V (2011). Pyrethroid resistance in African anopheline mosquitoes: what are the implications for malaria control?. Trends Parasitol.

[5] Lwetoijera DW, Harris C, Kiware SS, Dongus S, Devine GJ, McCall PJ (2014). Increasing role of *Anopheles funestus* and *Anopheles arabiensis* in malaria transmission in the Kilombero Valley, Tanzania. Malar J.

[6] Sougoufara S, Ottih EC, Tripet F (2020). The need for new vector control approaches targeting outdoor biting anopheline malaria vector communities. Parasites Vectors.

[7] Hemingway J, Shretta R, Wells TNC, Bell D, Djimde AA, Achee N (2016). Tools and strategies for malaria control and elimination: what do we need to achieve a grand convergence in malaria?. Plos Biol.

[8] Deredec A, Godfray HCJ, Burt A (2011). Requirements for effective malaria control with homing endonuclease genes. Proc Natl Acad Sci USA.

[9] Flores HA, O’Neill SL (2018). Controlling vector-borne diseases by releasing modified mosquitoes. Nat Rev Microbiol.

[10] Kyrou K, Hammond AM, Galizi R, Kranjc N, Burt A, Beaghton AK (2018). A CRISPR-Cas9 gene drive targeting doublesex causes complete population suppression in caged *Anopheles gambiae* mosquitoes. Nat Biotechnol.

[11] Champer J, Buchman A, Akbari OS (2016). Cheating evolution: engineering gene drives to manipulate the fate of wild populations. Nat Rev Genet.

[12] Brossard D, Belluck P, Gould F, Wirz CD (2019). Promises and perils of gene drives: navigating the communication of complex, post-normal science. Proc Natl Acad Sci USA.

[13] Aldersley A, Pongsiri A, Bunmee K, Kijchalao U, Chittham W, Fansiri T (2019). Too “sexy” for the field? Paired measures of laboratory and semi-field performance highlight variability in the apparent mating fitness of *Aedes aegypti* transgenic strains. Parasites Vectors.

[14] Leftwich PT, Bolton M, Chapman T (2016). Evolutionary biology and genetic techniques for insect control. Evol Appl.

[15] Soma DD, Maiga H, Mamai W, Bimbile-Somda NS, Venter N, Ali AB (2017). Does mosquito mass-rearing produce an inferior mosquito?. Malar J.

[16] Pennetier C, Warren B, Dabire KR, Russell IJ, Gibson G (2010). “Singing on the wing” as a mechanism for species recognition in the malarial mosquito *Anopheles gambiae*. Curr Biol.

[17] Clements A (2011). The biology of mosquitoes.

[18] Aldersley A, Cator LJ (2019). Female resistance and harmonic convergence influence male mating success in *Aedes aegypti*. Sci Rep.

[19] Manoukis NC, Diabate A, Abdoulaye A, Diallo M, Dao A, Yaro AS (2009). Structure and dynamics of male swarms of *Anopheles gambiae*. J Med Entomol.

[20] Belton P (1994). Attraction of male mosquitos to sound. J Am Mosquito Contr.

[21] Albert Joerg T, Kozlov AS (2016). Comparative aspects of hearing in vertebrates and insects with antennal ears. Curr Biol.

[22] Andres M, Seifert M, Spalthoff C, Warren B, Weiss L, Giraldo D (2016). Auditory efferent system modulates mosquito hearing. Curr Biol.

[23] Su MP, Andrés M, Boyd-Gibbins N, Somers J, Albert JT (2018). Sex and species specific hearing mechanisms in mosquito flagellar ears. Nature Commun.

[24] Fraenkel G (1932). Untersuchungen über die Koordination von Reflexen und automatisch-nervösen Rhythmen bei Insekten. Zeitschr Vgl Physiol.

[25] Villarreal SM, Winokur O, Harrington L (2017). The impact of temperature and body size on fundamental flight tone variation in the mosquito vector *Aedes aegypti* (Diptera: Culicidae): implications for acoustic lures. J Med Entomol.

[26] Cator LJ, NgʼHabi KR, Hoy RR, Harrington LC (2010). Sizing up a mate: variation in production and response to acoustic signals in *Anopheles gambiae*. Behav Ecol.

[27] Pantoja-Sanchez H, Gomez S, Velez V, Avila FW, Alfonso-Parra C (2019). Precopulatory acoustic interactions of the New World malaria vector *Anopheles albimanus* (Diptera: Culicidae). Parasites Vectors.

[28] Andrés M, Su MP, Albert J, Cator LJ (2020). Buzzkill: targeting the mosquito auditory system. Curr Opin Insect Sci.

[29] Stone CM, Tuten HC, Dobson SL (2013). Determinants of male *Aedes aegypti* and *Aedes polynesiensis* (Diptera: Culicidae) response to sound: efficacy and considerations for use of sound traps in the field. J Med Entomol.

[30] Sawadogo SP, Costantini C, Pennetier C, Diabate A, Gibson G, Dabire RK (2013). Differences in timing of mating swarms in sympatric populations of *Anopheles coluzzii* and *Anopheles**gambiae* s.s. (formerly *An*. *gambiae* M and S molecular forms) in Burkina Faso, West Africa. Parasites Vectors.

[31] Simoni A, Hammond AM, Beaghton AK, Galizi R, Taxiarchi C, Kyrou K (2020). A male-biased sex-distorter gene drive for the human malaria vector *Anopheles gambiae*. Nat Biotechnol.

