# Supplementary figures and images for: Assessing the acoustic behaviour of Anopheles gambiae (s.l.) dsxF mutants: implications for vector control
Source: Parasit Vectors. 2020 Oct 7;13:507. doi: 10.1186/s13071-020-04382-x (PMC7539510; doi:10.1186/s13071-020-04382-x)

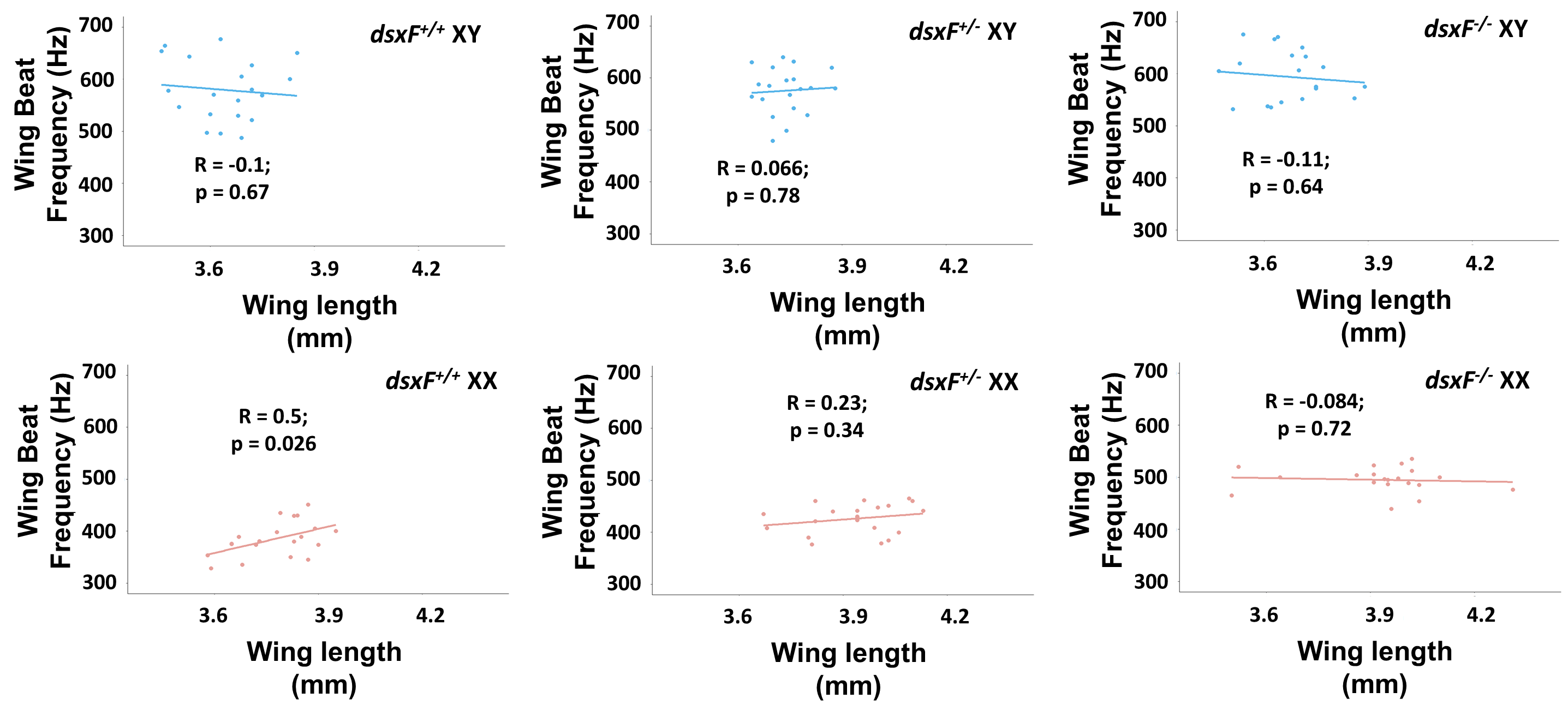

Supplement: Supplementary file 1 — Additional file 1: Figure S1. Phonotactic response of dsxF−/− males to phonotactic stimulation.Adjusted proportion of control mosquitoes responding to each stimulus type (380 Hz, 432 Hz and 497 Hz, respectively) for dsxF−/− XY mosquitoes. Centre line, median; error bars represent ± SE. [file 13071_2020_4382_MOESM1_ESM.tiff]

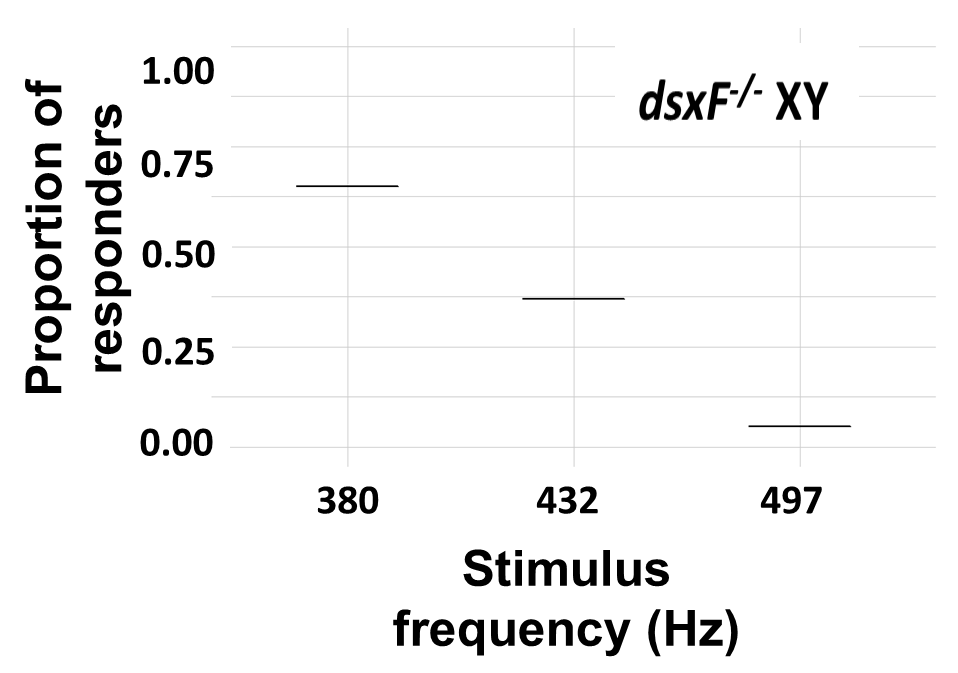

Supplement: Supplementary file 2 — Additional file 2: Figure S2. Correlations between wing length and wing beat frequency. Correlations between wing length (mm) and wing beat frequency (Hz) for all groups tested. Sample sizes are the same as for wing beat frequency calculations. [file 13071_2020_4382_MOESM2_ESM.tiff]
