# Supplementary material for: Assessing the acoustic behaviour of Anopheles gambiae (s.l.) dsxF mutants: implications for vector control
Source: Parasit Vectors. 2020 Oct 7;13:507. doi: 10.1186/s13071-020-04382-x (PMC7539510; doi:10.1186/s13071-020-04382-x)
Supplement: Supplementary file 3 — Additional file 3: Table S1. Outputs of linear model relating wing beat frequency to sex, genotype and wing length. Parameter estimates for linear model fitted in R using the lme4 package as: Wing beat frequency ~ Sex*Genotype + Wing length. Significant values are italicised. Whilst we found that sex, genotype, and sex:genotype were all highly significant factors in determining wing beat frequency, wing length was not found to significantly affect wing beat frequency. [file 13071_2020_4382_MOESM3_ESM.docx]

**Additional file 3: Table S1. Outputs of linear model relating wing beat frequency to sex, genotype and wing length.**

| **Variable** | **Estimate** | **SE** | **T value** | **Pr(>\|t\|)** |
| --- | --- | --- | --- | --- |
| (Intercept) | 335.44 | 118.73 | 2.825 | *0.00559 *** |
| Sex (Male) | 196.10 | 13.88 | 14.130 | *<2e-16 **** |
| Genotype (Heterozygous) | 39.06 | 14.16 | 2.758 | *0.00679 *** |
| Genotype (Homozygous) | 108.00 | 14.09 | 7.663 | *6.80e-12 **** |
| Wing Length | 13.16 | 31.36 | 0.420 | 0.67554 |
| Sex (Male): Genotype (Heterozygous) | -44.19 | 18.84 | -2.346 | *0.02072 ** |
| Sex (Male): Genotype (Homozygous) | -93.51 | 19.16 | -4.881 | *3.49e-06 **** |

Parameter estimates for linear model fitted in R using the lme4 package as:

Wing beat frequency ~ Sex*Genotype + Wing length. Significant values are italicised. Whilst we found that sex, genotype, and sex:genotype were all highly significant factors in determining wing beat frequency, wing length was not found to significantly affect wing beat frequency.
